# Single-Cell Transcriptome Analysis of Human Adipose-Derived Stromal Cells Identifies a Contractile Cell Subpopulation

**DOI:** 10.1155/2021/5595172

**Published:** 2021-04-28

**Authors:** Elize Wolmarans, Juanita Mellet, Chrisna Durandt, Fourie Joubert, Michael S. Pepper

**Affiliations:** ^1^Institute for Cellular and Molecular Medicine, Department of Immunology, SAMRC Extramural Unit for Stem Cell Research and Therapy, Faculty of Health Sciences, University of Pretoria, Pretoria 0001, South Africa; ^2^Centre for Bioinformatics and Computational Biology & Genomics Research Institute, University of Pretoria, Pretoria 0001, South Africa

## Abstract

The potential for human adipose-derived stromal cells (hASCs) to be used as a therapeutic product is being assessed in multiple clinical trials. However, much is still to be learned about these cells before they can be used with confidence in the clinical setting. An inherent characteristic of hASCs that is not well understood is their heterogeneity. The aim of this exploratory study was to characterize the heterogeneity of freshly isolated hASCs after two population doublings (P2) using single-cell transcriptome analysis. A minimum of two subpopulations were identified at P2. A major subpopulation was identified as contractile cells which, based on gene expression patterns, are likely to be pericytes and/or vascular smooth muscle cells (vSMCs).

## 1. Introduction

Human adipose-derived stromal cells (hASCs) have gained increasing attention as a potential cell therapy product. hASCs are classified as multipotent, fibroblast-like, plastic adherent cells that can easily be expanded in vitro into adipocytes, osteoblasts, and chondrocytes [1, 2]. A distinct advantage of hASCs is that a relatively large number of cells can be extracted from the adipose tissue with minor donor site morbidity [2, 3]. This has sparked worldwide growth of a new research field and industry.

It is well known that the cellular fraction isolated from the adipose tissue (stromal vascular fraction; SVF) and the resultant hASCs grown in culture are heterogeneous [1, 2, 4]. It is also well established that the expansion of heterogeneous cell populations leads to clonal selection and a loss of diversity [5–7]. It is unclear however whether the heterogeneity of the SVF, or the loss thereof during expansion, contributes to or impedes the potential therapeutic use of hASCs. A great deal of effort is thus being made to better understand the heterogeneous nature of the isolated cell populations.

Transcriptome analysis enables a comprehensive understanding of the molecular mechanisms that lead to functional variation and heterogeneity of cell populations. The characterization of cells based on protein-coding ribonucleic acid (RNA) that will be translated into functional proteins allows researchers to look at the transcriptome as a precursor of the proteome. Until recently, most transcriptome work has been conducted on pooled populations of cells providing an average of gene expression levels across the cell types present [8]. This masks the uniqueness and heterogeneity of gene expression patterns in individual cells, resulting in cells that are abundant being studied predominantly, while rare cell populations remain poorly characterized [9]. Single-cell RNA sequencing (scRNA-Seq) technologies constitute a powerful tool to quantify intrapopulation heterogeneity by studying gene expression patterns in individual cells [10, 11]. To date, scRNA-Seq has been used to dissect various heterogeneous cell populations and complex tissues, such as bone marrow-derived mesenchymal stromal cells (BM-MSCs) [12] and cells of the hematopoietic lineage [13].

Various studies have previously explored the heterogeneity of human and mouse ASCs at a single-cell level; however, most of these studies focused on the SVF [14–16]. For certain clinical applications, the heterogeneous hASC population may have to be expanded ex vivo to obtain clinically relevant cell numbers. We have thus undertaken an exploratory study to characterize cellular heterogeneity in freshly isolated, cultured hASCs at P2, using scRNA-Seq analysis.

## 2. Materials and Methods

### 2.1. Tissue Collection

Approval for this study was obtained from the Research Ethics Committee of the Faculty of Health Sciences, University of Pretoria (Reference number: 241/2015). Adipose tissue was obtained from healthy donors undergoing liposuction procedures, who had given written informed consent. Each donor was assigned a unique reference number to ensure anonymity. All donor information was kept confidential.

### 2.2. Isolation and Maintenance of Adipose-Derived Stromal Cells

Adipose tissue was processed using an adaptation of the Coleman method as published elsewhere [17]. The resulting SVF was resuspended in complete growth medium (CGM) consisting of Dulbecco's Modified Eagle's Medium (DMEM), 10% fetal bovine serum (FBS), and 1% penicillin/streptomycin (p/s). Cells were counted and seeded in CGM at a density of 5000 cells per cm^2^ in 25 or 75 cm^2^ culture flasks. Three hASC cultures (A20, A28 and A10) were isolated and maintained at 37°C, 5% CO_2_ in CGM. Cultures were expanded ex vivo for two rounds (P2). Cells were dissociated [using 3 mL 0.25% trypsin-ethylenediaminetetraacetic acid (EDTA) for 7 min at 37°C] when they reached confluence and reseeded at a density of 5000 cells/cm^2^.

### 2.3. Immunophenotyping of Human Adipose-Derived Stromal Cells

Flow cytometric analysis was used to determine immunophenotype before the start of each experiment. A six-color antibody panel was designed in-house based on the published criteria for ASCs [1, 18]. The following antibodies were included in the panel: CD34-PE-Cy7 (Clone 581; Beckman Coulter, Miami, USA), CD36-APC (Clone 5-271; Biolegend, California, USA), CD44-APC-Cy7 (Clone IM7; Biolegend, California, USA), CD45-Brilliant Violet™ (BV)510 (Clone HI30; BD Biosciences, New Jersey, USA), CD90-BV421 (Clone 5E10; BD Biosciences, New Jersey, USA), and CD105-PE (Clone 43A3; Beckman Coulter, Miami, USA).

Antibodies were added to 1 × 10^5^ cells from each cell suspension (5 *μ*L of each antibody), placed in a flow cytometry tube (FCT) and incubated at room temperature (RT) in the dark for 20 min. A second FCT containing 1 × 10^5^ unstained cells was prepared as a negative control and was used to set the negative/positive boundaries. After the incubation period, 1 mL phosphate buffered saline (PBS) containing 2% p/s was added to the aliquot. The sample was centrifuged at 300 g for 5 min, and the resulting supernatant was removed. The samples were phenotypically characterized using a Gallios flow cytometer (Figure S1 and Table S1; Beckman Coulter, Miami, USA). A total of 18 000 to 18 500 events were acquired for each sample and analyzed using Kaluza flow cytometry analysis software (version 2.1; Beckman Coulter, Miami, USA). Single-color FCTs (i.e., cells stained with only one monoclonal antibody per tube) were used to calculate the color compensation matrix in order to correct for spectral spill over into other detector channels.

### 2.4. Sorting of Human Adipose-Derived Stromal Cells

Sorting was done to ensure that only single, viable hASCs < 25 *μ*M in diameter, i.e., that were not too big for the microfluidic channels of the C1™ Single-Cell Auto Prep Array Integrated Fluidics Circuit (IFC) plates (Fluidigm, California, USA) were selected for analysis. hASC markers, CD90, and CD44 were included in the sort criteria to ensure inclusion of most hASC subpopulations at P2. CD45, a common leukocyte marker, was included to ensure that hematopoietic cells were excluded from the sort.

The sample was centrifuged at 300 g for 5 min at RT, the supernatant was removed, and 5 *μ*L of each antibody together with a viability marker (Propidium iodide; PI) was added to the sample. Viability ranged between 96 and 100%. The sample was incubated for 20 min at RT, protected from light. After the incubation period, the sample was washed with 1 mL PBS containing 2% p/s. The sample was centrifuged at 300 g for 5 min at RT, and the supernatant was removed. The sample was resuspended in 1 mL CGM for sorting using the FACSAria™ Fusion cell sorter (BD Biosciences, New Jersey, USA).

A sequential gating strategy was used to sort hASCs (see Figure S2). A side scatter (SS) vs. forward scatter (FS) two-parameter dot plot was used to exclude debris. Larger cells with stronger FS signals were also excluded due to the risk of potentially blocking the microfluidic channels of the IFC plate (Figure S2(a)). The SS area vs. SS width dot plot was used to exclude aggregates (Figure S2(b)), ensuring sorting of single cells. Viable cells were included based on negative PI staining (Figure S2(c)). Cells negative for CD45 and positive for CD44 and CD90 were included in the sort criteria (Figure S2(d)–(e)). The objective behind the gating strategy was thus to ensure that only single, viable, CD90^+^, CD44^+^, and CD45^–^ cells were selected and sorted.

The sort was performed using a neutral density filter of 2 and a nozzle size of 130 *μ*m. Sort purity was set on “Purity.” Cells (300 000) were sorted into preprepared sort tubes containing 2 mL CGM. After the sort, the samples were centrifuged, and the supernatant was removed. The sorted cells were resuspended in 500 *μ*L PBS (resulting concentration = 600 cells/*μ*L).

### 2.5. Single-Cell Capture, Lysis, Reverse Transcription, and Amplification

The Fluidigm C1™ Single-Cell Auto Prep System (Fluidigm, California, USA) together with IFC plates (17 to 25 *μ*m in size) were used to capture single cells. The Clontech® SMARTer® technology (Takara Bio, USA, Inc.) was used for the lysis, reverse transcription, and amplification of the resulting complementary DNA (cDNA), as per the manufacturer's instructions.

### 2.6. Library Preparation and Sequencing

Library preparation and sequencing were done by the Beijing Genomics Institute (BGI), Tai Po, Hong Kong, China.

### 2.7. Bioinformatic Pipeline

Analysis of the scRNA-Seq data was done using the RNA-Seq protocol of BCBIO, a bioinformatic-specific pipeline designed for automated, high throughput sequencing analysis (https://github.com/chapmanb/bcbio-nextgen). Data quality was determined using the FASTQC program, and the samples were aligned to the human reference genome (GRCh38) using HISAT2 (hierarchical indexing for spliced alignment of transcripts) [19]. Gene quantification was performed using featureCounts [20].

### 2.8. Quality Control Processing of the Single-Cell Data

Quality control metrics were applied to identify and remove poor quality samples from downstream analysis. A MultiQC report which can be viewed at http://wiki.bi.up.ac.za/scasc/multiqc was used to assess the quality of the data (Phred scores), the number of reads per sample, and the percentage alignment to the human reference genome (GRCh38). Cells with a Phred score < 30, reading depth of ≤ 100 000, or < 70% alignment to the human reference genome were removed from downstream analysis. Genes not expressed by three or more cells were removed from further analysis. Cells expressing < 200 genes were also removed. Cells expressing ≥ 10 000 genes were considered artifactual and were also removed. Further data analysis was done in *R* (version 3.5.2) [21] and RStudio (version 1.0.153) using the Seurat package (version 2.3.3) [22].

High levels of mitochondrial gene expression are usually indicators of cellular stress [23]; thus, samples containing > 10% mitochondrial genes were excluded from downstream analysis. Mitochondrial genes were also regressed out of the single-cell data due to possible interference in differential gene expression analysis. Various mitochondrial pseudogenes (*MTND1P23*, *MTND2P28*, *MTCO1P12*, *MTCO2P12*, *MTATP8P1*, *MTATP6P1*, *MTCO3P12*, and *MTCYBP45*) were highly expressed, which skewed the initial results and were therefore removed from downstream analysis.

Cell cycle genes were also regressed out of the dataset to prevent interference with downstream differential gene expression analysis [24].

Samples were normalized by employing global scale normalization (lognormalize). This function normalizes gene expression measurements for each cell to the total expression and multiplies this by a scale factor (10 000).

### 2.9. Batch Effect Correction Using Canonical Correlation Analysis

Initial clustering of the cells using the Seurat package resulted in clustering based on biological replicates (Figure S3), most likely due to biological and technical variability. A computational strategy introduced by Butler et al. (2018) referred to as canonical correlation analysis (CCA) was used to correct for this [22]. Heatmaps (Figure S4) and a biweight midcorrelation (bicor) saturation plot (Figure S5) were used to identify the most significant canonical correlation vectors (CCs) to align the three biological replicates for downstream analysis. Biological replicates (A20, A28, and A10) were aligned using 1000 genes with the highest dispersion in all three data sets, and six CCs were used for downstream clustering.

### 2.10. *t*-SNE Clustering Analysis

Clusters of transcriptomically similar cells were identified using a shared nearest neighbor (SNN) modularity optimization-based clustering algorithm [25], and nonlinear dimensional reduction (*t*-distributed stochastic neighbour embedding; *t*-SNE) was used to visualize possible clusters. A number of resolution parameters ranging from 0.5 to 1.2 were used. Differentially expressed genes (DEGs) specific to each cluster were identified using the FindAllMarkers function.

### 2.11. Statistical Analysis

DEGs for each cluster were identified based on a log fold change (logFC) ≥ 0.5. Statistical significance was tested using the nonparametric Wilcoxon rank sum test with Bonferroni correction. An adjusted *P* value of *α* ≤ 0.01 was considered significant.

## 3. Results

**Figure 1:**
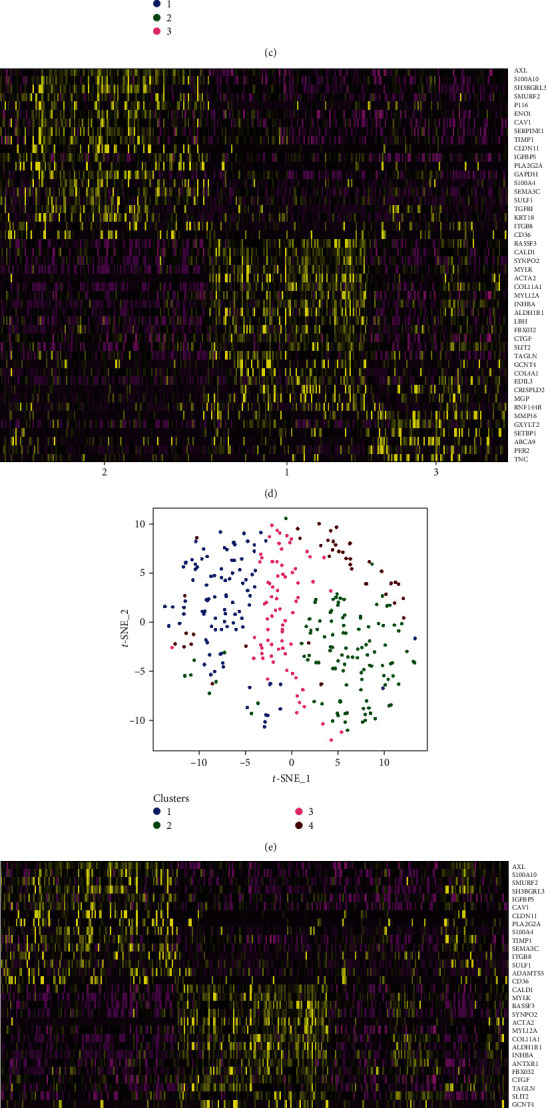
*t*-SNE plots illustrating the different cluster number permutations and heatmaps illustrating the top DEGs for each cluster. The colored dot clouds on the tSNE plots represent individual clusters. (a) Two clusters were identified at resolution 0.5 while (c) three clusters were identified at resolutions 0.6 and 0.7. (e) Four were identified at resolution 0.8 and (g) resolution 0.9 to 1.2 consistently identified five clusters. For each cluster number permutation, there is a corresponding heatmap illustrating the top DEGs per cluster (two, three, four, and five clusters, respectively). Yellow represents highly expressed genes while purple represents poorly expressed genes.

A minimum of two and a maximum of five clusters were identified in the hASCs at P2. While identifying clusters, it is possible to change the clustering resolution. Increasing resolution usually results in an increased number of clusters identified. Two clusters were identified at resolution 0.5 (Figure 1(a)), while three clusters were identified at resolutions 0.6 and 0.7 (Figure 1(c)). Four clusters were identified at resolution 0.8 (Figure 1(e)) and resolutions 0.9 to 1.2 consistently identified five clusters (Figure 1(g)). DEGs were identified for each cluster number permutation (two to five clusters). The genes identified and reported were based on a positive logFC of ≥ 0.5, with a *P* value of ≤ 0.01. However, only genes with an adjusted *P* value of ≤ 0.01 were considered for further analysis. Figures 1(b), 1(d), 1(f), and 1(h) show heatmaps illustrating the DEGs for each cluster of the various cluster permutations identified (two, three, four and five clusters, respectively). The list of statistically significant DEG (*α* ≤ 0.01) can be viewed in Table S2.

**Figure 2:**
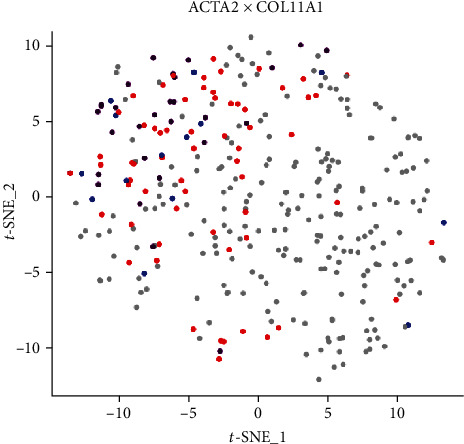
*t*-SNE plot illustrating the expression of *ACTA2* and *COL11A1* in individual hASCs. Each dot on the plot represents an individual cell. Cells expressing *ACTA2* are represented as blue dots while cells expressing *COL11A1* are represented as red dots. Cells expressing both are colored purple while the grey dots represent cells that express neither of the selected genes. The data shows the selected genes being predominantly expressed in cluster 1 cells.

Cluster 1 remained stable for resolutions 0.6 to 1.2. The list of statistically significant (*α* ≤ 0.01) DEGs also remained similar in this cluster across the different cluster number permutations (Table 1). Of these genes, two were consistently identified with a positive logFC of ≥ 1 for all the different cluster number permutations, namely, *ACTA2* and *COL11A1*. *COL11A1* codes for the proalpha1 (XI) chain of type XI collagen [26]. *ACTA2* codes for smooth muscle alpha-actin protein and is one of the best known molecular markers for contractile cells such as vascular smooth muscle cells (vSMCs) and pericytes [27–30]. The t-SNE plot in Figure 2 shows the expression pattern of both *ACTA2* and *COL11A1* in single hASCs and clearly shows that these genes are predominantly expressed in cells of cluster 1. Upon further investigation, other statistically significant DEGs were identified within cluster 1 that also encode for proteins involved in contractile cell structure and function. A list of these genes, the proteins they encode and their respective functions, are shown in Table 1.

Kumar et al. (2017) used bulk RNA-Seq techniques to study pericytes and vSMCs that were differentiated from mesenchymangioblasts derived from human-induced pluripotent stem cells (iPSCs) [28]. Their results showed that vSMCs and pericytes share expression of *ACTA2*, *TAGLN*, *TPM2*, and *MYLK,* while *MYL12A*, *CALD1*, *COL11A1*, and *SYNPO2* were only identified in vSMCs [28]. Based on the list of statistically significant DEGs (Table 1), it is hypothesized that cluster 1 is comprised of contractile cells including pericytes and/or vSMCs.

Cluster 2 remained stable at low resolutions (0.5 to 0.7) but became fragmented when the resolution was increased to 0.8. The additional clusters identified at the higher resolutions (clusters 4 and 5) showed very few DEGs and overlapping DEGs with cluster 2 at lower resolutions. Based on these observations, clusters 4 and 5 cannot conclusively be identified as separate subpopulations within this dataset.

Even though cluster 3 remained stable for most of the cluster number permutations (resolutions 0.6 to 1.2), only three DEGs were identified to be statistically significant (*α* ≤ 0.01) in this cluster: *MMP16*, *SFRP2*, and *SOX4*. The lack of significant DEGs in this cluster did not allow for clear identification of the cells and/or their function within this cluster.

## 4. Discussion

hASCs have shown great promise as a potential cellular therapeutic product for a number of diseases, and efforts are being made to better understand the heterogeneous nature of the isolated cell populations. Single-cell transcriptome technologies are powerful tools for studying cellular heterogeneity. Very few single-cell studies on hASCs have been published to date, and the majority have focused on the SVF. There is however little consensus on the number of subpopulations present in the SVF or the specific markers for each subpopulation. This is mainly due to different aims and methodologies employed by each research group. Rennert et al. (2016) studied 96 genes using single cells of human SVF and identified up to five subpopulations [14]. Hardy et al. (2017) also used single-cell technology to study two subpopulations in SVF, named adventitial stromal cells and pericytes, based on the following phenotypic markers: CD31, CD45, CD146, and CD34 [15]. Pericytes had a transcriptomic signature distinct from adventitial stromal cells. Each subpopulation could be further divided into aldehyde dehydrogenase (ALDH)-bright and ALDH-dim populations [15]. It was also reported that a minimum of six genes could be used to discriminate between pericytes and adventitial stromal cells [15].

Clonal expansion in primary cultures such as ASCs has been well documented [5–7, 36]. In an elegant study conducted by Selich et al. (2016) on MSCs derived from the umbilical cord, it was shown that the first severe loss of diversity in primary cell cultures is observed within the first three passages [5]. The second drop in clonal diversity could be observed during further expansion as specific clones become dominant [5].

The cellular transcriptomic heterogeneity of hASCs cultured in vitro has not been comprehensively investigated at the single-cell level. In our study, the aim was to characterize heterogeneity of freshly isolated hASCs that had undergone two population doublings (P2; before severe loss of diversity). The objectives of this study were (a) to determine the number of subpopulations present in hASCs at P2 via clustering and (b) to identify statistically significant DEGs for each cluster.

hASCs were sourced from three female donors. The percentage of hASCs that expressed phenotypic markers was between 88 and 92% at P2. It is well documented that differentiation and proliferation potential of hASC decreases in older females; however, the hASC surface marker expression remains intact [37, 38].

Clustering was done at different clustering resolutions. hASCs were sourced from three different donors, and there was the possibility of clonal selection during expansion. However, scRNA-Seq analysis of 322 cells revealed limited heterogeneity with only two to five subpopulations present at P2. Two clusters remained consistent at resolutions 0.6 to 1.2, namely, clusters 1 and 3. Even though cluster 3 was consistently identified throughout, very few statistically significantly (*α* ≤ 0.01) DEGs could be identified for this cluster. In contrast, the list of statistically significant (*α* ≤ 0.01) DEGs (in particular, *ACTA2*, *COL11A1*, *SYNPO2*, *TAGLN*, *CALD1*, *MYLK*, *MYL12A*, and *TPM2*) identified for cluster 1 suggests that this subpopulation is comprised of contractile cells such as pericytes and/or vSMCs.

Pericytes and vSMCs are collectively known as perivascular cells. Their main function is to support and stabilize vascular networks and to regulate blood flow [4, 39]. vSMCs form the media of arteries and veins of varying complexity and are also found in the adventitia of large veins, while pericytes surround smaller blood vessels such as capillaries and postcapillary venules [27, 39, 40]. Both cell types are believed to be present in the SVF of the isolated adipose tissue [1, 2, 4]. A single-cell study by Hardy et al. (2017) determined that pericytes (identified based on sorting criteria) had a distinct transcriptomic signature that could be differentiated from the rest of the SVF using a minimum of six distinct molecular markers. One of these markers was *ACTA2* [15], which is one of the main molecular markers identified in cluster 1. In addition, Baryawno et al. (2019) did a comprehensive single-cell study on murine BM-MSCs. They identified a dominant subpopulation expressing *ACTA2* which they classified as pericytes [12]. *ACTA2* appears however to be expressed by both pericytes and vSMCs [30, 31]. In a recent study, Kumar et al. (2017) studied pericytes and vSMCs differentiated from mesenchymangioblasts [28]. Transcriptome data from that study revealed that vSMCs and pericytes share the expression of *ACTA2*, *TAGLN*, *TPM2*, *MYLK* and *MYL12A*, while *CALD1*, *COL11A1*, and *SYNPO2* that were only identified in vSMCs [28]. It is therefore hypothesized that cluster 1 cells are pericytes and/or vSMCs.

We acknowledge that the transcriptome is only a precursor to translated proteins and therefore, its analysis in isolation has limitations with regard to functional relevance. It would therefore be useful to confirm and distinguish between the different contractile cells; however, there is currently no single marker that can be used to distinguish between vSMCs and pericytes [39, 41], and an array of markers is therefore used. Many membrane-specific markers have been identified for pericytes which include CD146, neuron-glial antigen 2 (NG2), and ribosomal protein S14 (3G5) [27, 28]. vSMCs are usually identified via immunohistochemistry using proteins such as myocardin, transgelin, and myosin heavy chain 11 [28, 30]. Identifying membrane-specific proteins will allow for the isolation of specific clusters of cells by FACS. Of particular interest would be the presence or absence of pericytes within cluster 1. Pericytes have been reported by multiple research groups to be potential progenitor cells with the ability to differentiate into osteocytes, adipocytes, chondrocytes, and myocytes [4, 42–46], an ability shared by hASCs [1, 47]. If cluster 1 could be separated from the rest of the hASC population, it would be of interest to see whether the differentiation capabilities usually observed in hASCs could be ascribed to pericytes, an as yet undiscovered progenitor/stem cell population, or both. The hypothesized contractile subpopulation constituted 31% of the 322 hASCs studied at P2. It will be important to determine to what extent the differentiation capabilities of pericytes contributes to the overall differentiation potential of the broader heterogeneous hASC cell population. Collagen gel contraction assays could be useful to further confirm the functional capabilities of these cells.

MSC populations exhibit considerable heterogeneity between donors and within individual donor populations, and the mechanisms that regulate self-renewal and lineage specification remain largely unexplored. This creates significant obstacles in research and in efforts aimed at developing clinical manufacturing protocols to produce standardized MSC therapeutic products [48]. The functional variation and heterogeneity of MSCs from various sources are purported to be the main reasons for inconsistent and controversial results observed in clinical trials [48, 49]. Single-cell sequencing technologies are powerful tools that can be used to explore the heterogeneity within cell populations. Traditional sequencing approaches analyze pooled populations which masks heterogeneity as a result of poorly visible smaller subpopulations. Single-cell RNA-Seq allows identification and transcriptomic characterization of large and small subpopulations which may lead to the discovery of specific markers relevant to therapeutic applications. Understanding the mechanisms that regulate fate specifications could lead to tailored expansion of specific lineages with unique biological properties suited for specific therapeutic needs.

Standardization of isolation and expansion methods is a key element in quality control of MSC products. We recognize that the use of FBS in our experiments is a confounding factor. These results form part of an initial study in our group to explore the transcriptomic heterogeneity of hASCs cultured in vitro. We realize that these experiments would need to be repeated using good manufacturing practices (GMP) conditions for better clinical translation.

Another limitation of this study is that the C1™ IFC plates can only capture cells of a specific size range (17 to 25 *μ*M). This leads to the noncapture of cells falling outside this size range. This creates a bias in the hASCs captured, which are usually quite heterogeneous in the cell size. Future experiments should therefore use a system that will allow capture of all cell sizes present.

## 5. Conclusions

In conclusion, this exploratory study has characterized the heterogeneity of hASCs at P2 using transcriptome analysis at the single-cell level. Our results reveal the presence of at least two subpopulations of cells. One of these subpopulations is believed to be contractile cells that include pericytes and/or vSMCs.

## Figures and Tables

**Table 1:** Statistically significant (*α* ≤ 0.01) DEGs identified in cluster 1 that encode for proteins that play a role in contractile cells.

Genes	Protein	Function	References
*ACTA2*	Alpha smooth muscle actin (*α*-SMA)	Involved in vascular contractibility and blood pressure homeostasis.	[15, 27–31]
*COL11A1*	Collagen alpha-1(XI) chain	One of three chains that make up type XI collagen.	[26, 28]
*SYNPO2*	Myopodin or synaptopodin-2	Nonmotor actin binding protein that interacts with *α*-SMA.	[28]
*CALD1*	Caldesmon (heavy and light chain)	Cytoskeletal protein that interacts with actin, tropomyosin, myosin, calmodulin, and phospholipids.	[28, 30–32]
*TAGLN*	Transgelin (SM22-*α*)	Nonmotor actin binding protein.	[27, 28, 31, 33]
*MYLK*	Myosin light chain kinase	Calcium/calmodulin dependent enzyme that phosphorylates myosin regulatory light chains.	[28, 34]
*MYL12A*	Myosin regulatory light chain 12A	Regulates smooth muscle contraction.	[28, 35]
*TPM2*	Beta-tropomyosin	Controls the binding of myosin and actin.	[28, 34]

## Data Availability

The single-cell RNA-Seq data used to support the findings of this study are available from the corresponding author upon request.

## Supplementary Materials

Supplementary Materials Is included as a separate file and includes the following figures and tables: Figure S1: representative dot plots illustrating the sequential gating strategy used to study the combined immunophenotypic profile of hASCs. The hASC population of interest was visualized on an FS vs. SS log two-parameter dot plot. (a) Single hASCs were included, and debris was excluded by gating around intact cells. The gated hASCs were then plotted on two-parameter dot plots, allowing for the simultaneous analysis of expression of two surface markers. (b) hASCs were identified that were positive for CD44 and CD90 (quadrant A++). (c) CD105 positive and CD45 negative (quadrant B-+). The final plot represents (d) CD44^+^/CD90^+^/CD105^+^/CD45^–^ hASCs stained for CD36 and CD34. This figure is relevant to the Materials and Methods section. Also see Table S2. Figure S2: an example of the sequential gating strategy for the sorting of the hASCs. (a) SS vs. FS two-parameter dot plot was used to identify hASCs (region A) and exclude debris (region F). (b) SS area vs. SS width dot plot, gated on A, was used to exclude small clumps of cells, ensuring that only single cells (region B) are included in the downstream sorting region. (c) A PI vs. count histogram, gated on B, was used to select for viable cells (region C). Selection of the ASC population was further refined in the next two plots. First, a CD44 APC-Cy7/CD45 KO plot that was gated on viable cells was used. (d) Only cells positive for the CD44 and negative for CD45 were selected. The final selection for sorting was based on (e) positive staining of ASCs for CD44 and CD90, using a CD44 APC-Cy7 vs. CD90 PE-Cy5 plot. The logic behind the sequential gating strategy was to ensure that only single, viable, CD90^+^, CD44^+^, and CD45^–^ cells were selected and sorted. This figure is relevant to the Materials and Methods section. Figure S3: *t*-SNE plot illustrating the batch effect observed during initial single-cell analysis using the Seurat package. The different colored dot clouds represent individual clusters of cells. During preliminary clustering, it was noticed that the cells were clustering according to their original identity, referring to the cell cultures from which they were originally isolated. This figure is relevant to the Materials and Methods section. Figure S4: heatmaps illustrating the top 20 DEGs for the first 12 CCs. The heatmaps show the top 20 DEGs (rows) identified for each cell (columns) in every CC. Yellow represents genes with positive scores (highly expressed genes) while purple represents negative scores (low level expressed genes). DEGs could be visualized (yellow and purple blocks) for up to 9 CCs. From 10 CCs onward, no distinct pattern could be distinguished. This indicates that CCs 1 to 9 could be used for downstream analysis. This figure is relevant to the Materials and Methods section. Figure S5: biweight midcorrelation (bicor) saturation plot illustrating the smoothed shared correlation strength versus CCs between the different datasets. The plot measured the correlation strength for 15 CCs of the different datasets. Each colored line on the graph represents the Seurat object of the individual datasets, named according to the cell culture from which the cells originate. The graph shows a saturation point (where the curve in the graph starts to flatten) at 6 CCs. The first 6 CCs were used for downstream analyses. This figure is relevant to the Materials and Methods section. Table S1. List of statistically significant (*α* ≤ 0.01) DEGs identified in the different cluster number permutations. This table is related to Figure 1, Figure 2, and Table 1. Table S2: percentage of hASCs (P2) from the three cultures used during the single-cell experiments which adhered to the phenotype: CD44^+^/CD90^+^/CD105^+^/CD45^–^/CD34^–^/CD36^+^. All cultures adhered to the phenotypic criteria set out by the International Federation for Adipose Therapeutics and Science (IFATS) and the International Society for Cellular Therapy (ISCT). This table is relevant to the Material and Method section, and Figure S1.
