# Supplementary material for: Single-Cell Transcriptome Analysis of Human Adipose-Derived Stromal Cells Identifies a Contractile Cell Subpopulation
Source: Stem Cells Int. 2021 Apr 28;2021:5595172. doi: 10.1155/2021/5595172 (PMC8102097; doi:10.1155/2021/5595172)
Supplement: Supplementary Materials — Is included as a separate file and includes the following figures and tables: Figure S1: representative dot plots illustrating the sequential gating strategy used to study the combined immunophenotypic profile of hASCs. The hASC population of interest was visualized on an FS vs. SS log two-parameter dot plot. (a) Single hASCs were included, and debris was excluded by gating around intact cells. The gated hASCs were then plotted on two-parameter dot plots, allowing for the simultaneous analysis of expression of two surface markers. (b) hASCs were identified that were positive for CD44 and CD90 (quadrant A++). (c) CD105 positive and CD45 negative (quadrant B-+). The final plot represents (d) CD44+/CD90+/CD105+/CD45– hASCs stained for CD36 and CD34. This figure is relevant to the Materials and Methods section. Also see Table S2. Figure S2: an example of the sequential gating strategy for the sorting of the hASCs. (a) SS vs. FS two-parameter dot plot was used to identify hASCs (region A) and exclude debris (region F). (b) SS area vs. SS width dot plot, gated on A, was used to exclude small clumps of cells, ensuring that only single cells (region B) are included in the downstream sorting region. (c) A PI vs. count histogram, gated on B, was used to select for viable cells (region C). Selection of the ASC population was further refined in the next two plots. First, a CD44 APC-Cy7/CD45 KO plot that was gated on viable cells was used. (d) Only cells positive for the CD44 and negative for CD45 were selected. The final selection for sorting was based on (e) positive staining of ASCs for CD44 and CD90, using a CD44 APC-Cy7 vs. CD90 PE-Cy5 plot. The logic behind the sequential gating strategy was to ensure that only single, viable, CD90+, CD44+, and CD45– cells were selected and sorted. This figure is relevant to the Materials and Methods section. Figure S3: t-SNE plot illustrating the batch effect observed during initial single-cell analysis using the Seurat package. The diffe [file 5595172.f1.docx]

**Supplementary Material**


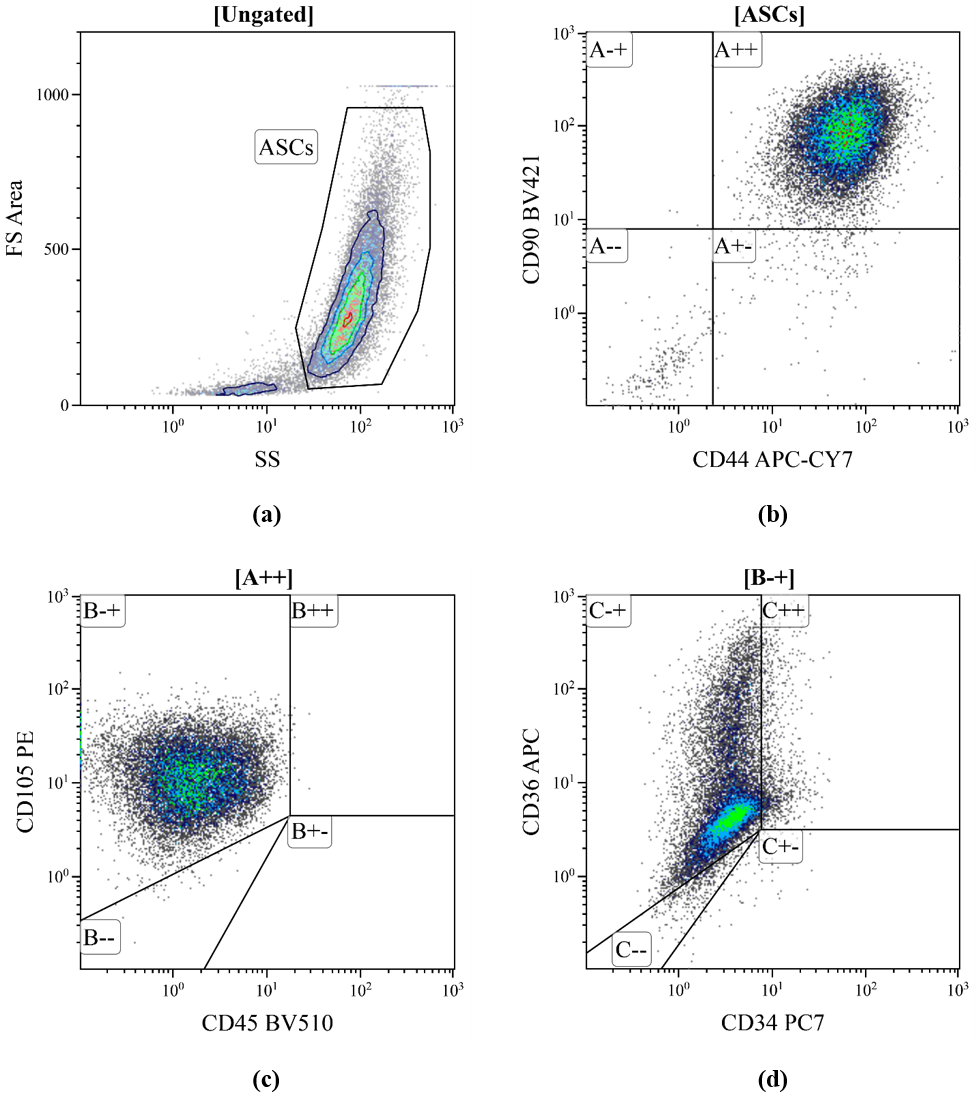


Figure S1: representative dot plots illustrating the sequential gating strategy used to study the combined immunophenotypic profile of hASCs. The hASC population of interest was visualized on an FS vs. SS log two-parameter dot plot. (a) Single hASCs were included, and debris was excluded by gating around intact cells. The gated hASCs were then plotted on two-parameter dot plots, allowing for the simultaneous analysis of expression of two surface markers. (b) hASCs were identified that were positive for CD44 and CD90 (quadrant A++). (c) CD105 positive and CD45 negative (quadrant B-+). The final plot represents (d) CD44^+^/CD90^+^/CD105^+^/CD45^–^ hASCs stained for CD36 and CD34. This figure is relevant to the Materials and Methods section. Also see Table S2.

**
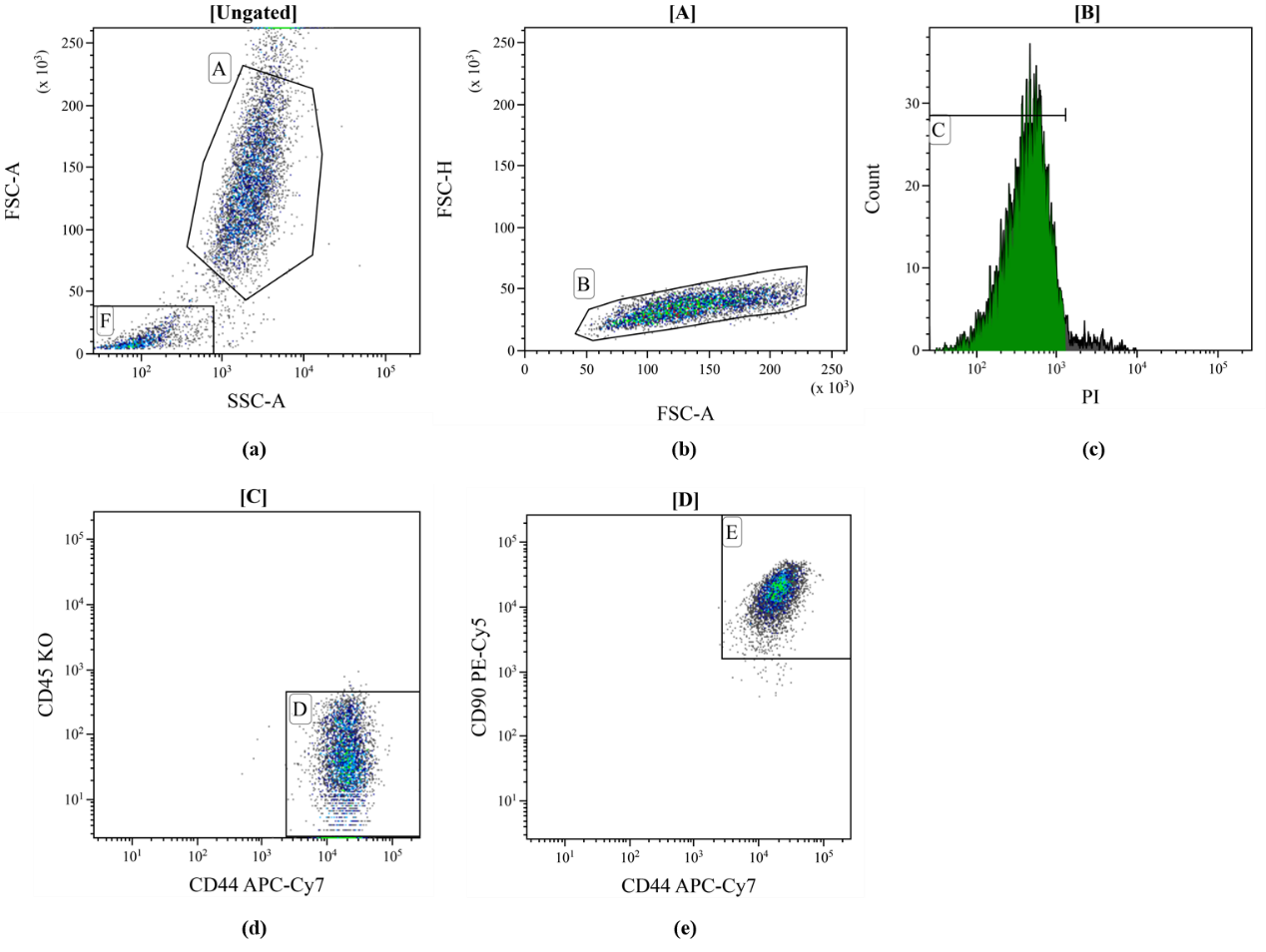
**

Figure S2: an example of the sequential gating strategy for the sorting of the hASCs. (a) SS vs. FS two-parameter dot plot was used to identify hASCs (region A) and exclude debris (region F). (b) SS area vs. SS width dot plot, gated on A, was used to exclude small clumps of cells, ensuring that only single cells (region B) are included in the downstream sorting region. (c) A PI vs. count histogram, gated on B, was used to select for viable cells (region C). Selection of the ASC population was further refined in the next two plots. First, a CD44 APC-Cy7/CD45 KO plot that was gated on viable cells was used. (d) Only cells positive for the CD44 and negative for CD45 were selected. The final selection for sorting was based on (e) positive staining of ASCs for CD44 and CD90, using a CD44 APC-Cy7 vs. CD90 PE-Cy5 plot. The logic behind the sequential gating strategy was to ensure that only single, viable, CD90^+^, CD44^+^ and CD45^–^ cells were selected and sorted. This figure is relevant to the Materials and Methods section.


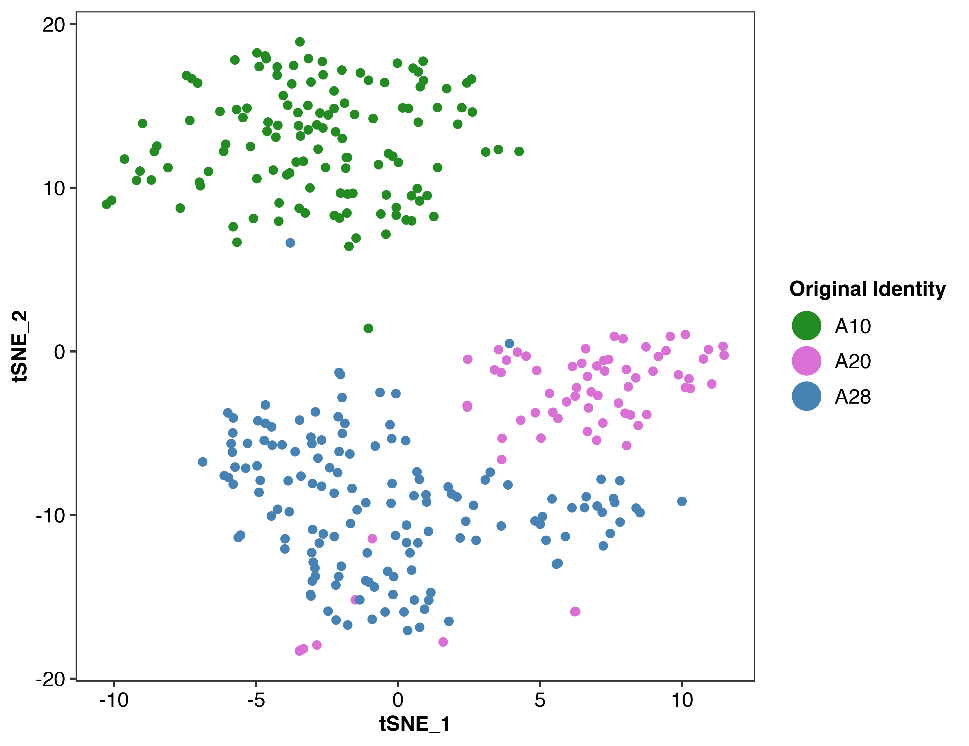


Figure S3: t-SNE plot illustrating the batch effect observed during initial single-cell analysis using the Seurat package. The different colored dot clouds represent individual clusters of cells. During preliminary clustering, it was noticed that the cells were clustering according to their original identity, referring to the cell cultures from which they were originally isolated. This figure is relevant to the Materials and Methods section.


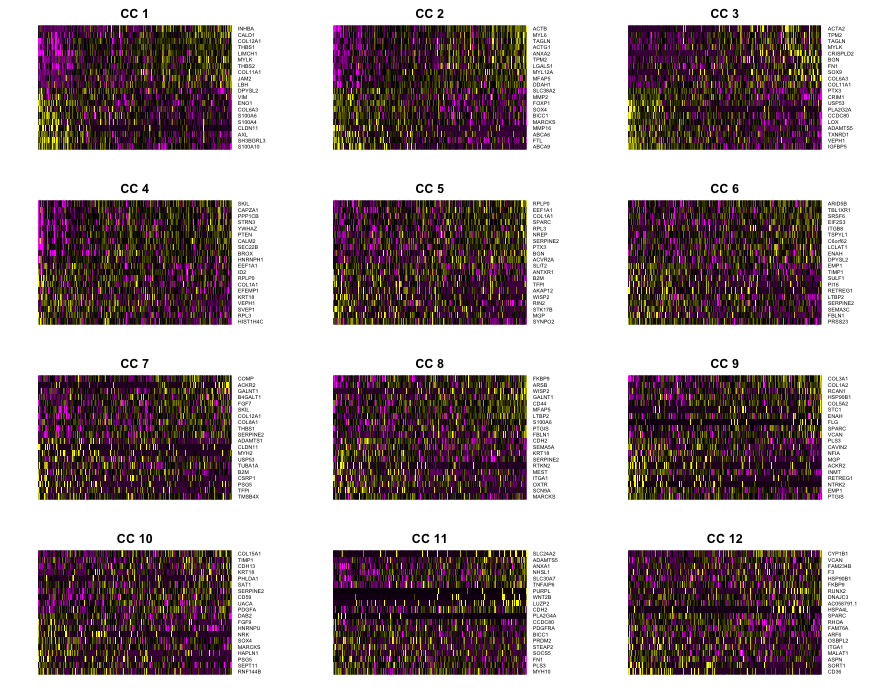
Figure S4: heatmaps illustrating the top 20 DEGs for the first 12 CCs. The heatmaps show the top 20 DEGs (rows) identified for each cell (columns) in every CC. Yellow represents genes with positive scores (highly expressed genes) while purple represents negative scores (low level expressed genes). DEGs could be visualized (yellow and purple blocks) for up to 9 CCs. From 10 CCs onward, no distinct pattern could be distinguished. This indicates that CCs 1 to 9 could be used for downstream analysis. This figure is relevant to the Materials and Methods section.


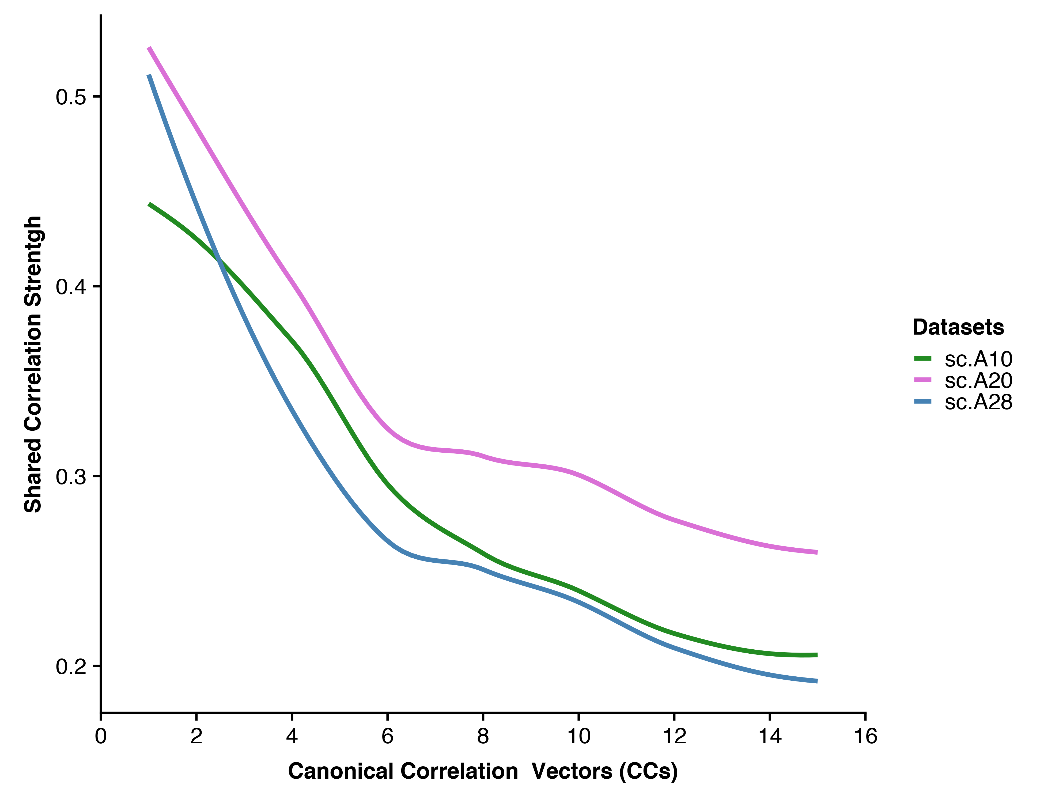


Figure S5: biweight midcorrelation (bicor) saturation plot illustrating the smoothed shared correlation strength versus CCs between the different datasets. The plot measured the correlation strength for 15 CCs of the different datasets. Each colored line on the graph represents the Seurat object of the individual datasets, named according to the cell culture from which the cells originate. The graph shows a saturation point (where the curve in the graph starts to flatten) at 6 CCs. The first 6 CCs were used for downstream analyses. This figure is relevant to the Materials and Methods section.

Table S1. List of statistically significant (α ≤ 0.01) DEGs identified in the different cluster number permutations. This table is related to Figure 1, Figure 2, and Table 1.

| **2 Clusters** | | | | **3 Clusters** | | | | **4 Clusters** | | | | **5 Clusters** | | | |
| --- | --- | --- | --- | --- | --- | --- | --- | --- | --- | --- | --- | --- | --- | --- | --- |
| **Gene** | **Cluster** | **Average LogFC** | **Adjusted P-Value** | **Gene** | **Cluster** | **Average LogFC** | **Adjusted P-Value** | **Gene** | **Cluster** | **Average LogFC** | **Adjusted P-Value** | **Gene** | **Cluster** | **Average LogFC** | **Adjusted P-Value** |
| *COL11A1* | 1 | 1.19 | 1.00e-18 | *RASSF3* | 1 | 0.65 | 1.27e-15 | *CALD1* | 1 | 0.54 | 2.81e-15 | *SYNPO2* | 1 | 0.82 | 1.06e-14 |
| *MYLK* | 1 | 0.91 | 1.28e-17 | *CALD1* | 1 | 0.54 | 1.65e-15 | *MYLK* | 1 | 0.92 | 4.83e-15 | *CALD1* | 1 | 0.54 | 1.62e-14 |
| *RASSF3* | 1 | 0.63 | 5.59e-16 | *SYNPO2* | 1 | 0.82 | 3.36e-15 | *RASSF3* | 1 | 0.64 | 7.07e-15 | *RASSF3* | 1 | 0.64 | 2.01e-14 |
| *INHBA* | 1 | 1.03 | 7.55e-15 | *MYLK* | 1 | 0.91 | 6.07e-15 | *SYNPO2* | 1 | 0.81 | 1.35e-14 | *MYLK* | 1 | 0.88 | 6.45e-14 |
| *SFRP2* | 1 | 0.74 | 4.04e-14 | *ACTA2* | 1 | 1.57 | 4.66e-13 | *ACTA2* | 1 | 1.59 | 5.19e-14 | *ACTA2* | 1 | 1.57 | 7.72e-14 |
| *SLIT2* | 1 | 0.67 | 1.05e-10 | *COL11A1* | 1 | 1.02 | 4.75e-13 | *MYL12A* | 1 | 0.55 | 3.03e-13 | *COL11A1* | 1 | 1.04 | 2.24e-12 |
| *FBXO32* | 1 | 0.86 | 1.68e-10 | *MYL12A* | 1 | 0.53 | 3.95e-12 | *COL11A1* | 1 | 1.04 | 1.03e-12 | *MYL12A* | 1 | 0.53 | 5.86e-12 |
| *ITGA8* | 1 | 0.58 | 6.88e-08 | *INHBA* | 1 | 0.88 | 4.59e-11 | *ALDH1B1* | 1 | 0.95 | 1.65e-10 | *INHBA* | 1 | 0.89 | 1.03e-10 |
| *CRISPLD2* | 1 | 0.79 | 7.86e-07 | *ALDH1B1* | 1 | 0.91 | 4.49e-09 | *INHBA* | 1 | 0.87 | 1.69e-10 | *ALDH1B1* | 1 | 0.93 | 6.31e-10 |
| *ANTXR1* | 1 | 0.51 | 1.92e-06 | *LBH* | 1 | 0.54 | 1.42e-08 | *ANTXR1* | 1 | 0.53 | 1.52e-08 | *ANTXR1* | 1 | 0.53 | 4.95e-08 |
| *EDIL3* | 1 | 0.80 | 3.27e-06 | *FBXO32* | 1 | 0.86 | 7.01e-08 | *FBXO32* | 1 | 0.84 | 9.44e-08 | *FBXO32* | 1 | 0.86 | 1.16e-07 |
| *ACTA2* | 1 | 1.25 | 4.23e-05 | *CTGF* | 1 | 0.60 | 2.75e-07 | *CTGF* | 1 | 0.58 | 3.11e-06 | *CTGF* | 1 | 0.59 | 1.33e-06 |
| *SYNPO2* | 1 | 0.56 | 4.04e-04 | *SLIT2* | 1 | 0.56 | 3.73e-06 | *TAGLN* | 1 | 0.65 | 1.08e-05 | *TAGLN* | 1 | 0.65 | 2.51e-05 |
| *MGP* | 1 | 0.59 | 6.25e-04 | *TAGLN* | 1 | 0.61 | 5.72e-05 | *SLIT2* | 1 | 0.54 | 1.10e-05 | *SLIT2* | 1 | 0.54 | 2.59e-05 |
| *AXL* | 2 | 0.84 | 1.04e-19 | *AXL* | 2 | 0.82 | 2.23e-18 | *GCNT4* | 1 | 0.90 | 3.14e-04 | *TPM2* | 1 | 0.54 | 9.01e-04 |
| *SH3BGRL3* | 2 | 0.90 | 7.46e-18 | *S100A10* | 2 | 0.76 | 7.23e-18 | *TPM2* | 1 | 0.53 | 8.57e-04 | *IGFBP5* | 2 | 1.12 | 6.58e-09 |
| *S100A10* | 2 | 0.73 | 3.08e-16 | *SH3BGRL3* | 2 | 0.92 | 1.28e-15 | *AXL* | 2 | 0.69 | 2.03e-12 | *VEPH1* | 2 | 0.67 | 2.31e-08 |
| *SMURF2* | 2 | 0.60 | 7.64e-11 | *SMURF2* | 2 | 0.64 | 5.32e-12 | *S100A10* | 2 | 0.64 | 2.31e-10 | *ADAMTS5* | 2 | 0.66 | 3.87e-04 |
| *SERPINE1* | 2 | 0.89 | 6.17e-10 | *PI16* | 2 | 0.88 | 3.99e-10 | *SMURF2* | 2 | 0.60 | 9.17e-09 | *SFRP2* | 3 | 0.55 | 5.70e-05 |
| *VEPH1* | 2 | 0.69 | 1.28e-09 | *ENO1* | 2 | 0.62 | 1.23e-09 | *SH3BGRL3* | 2 | 0.62 | 3.30e-08 | *MMP16* | 3 | 0.93 | 3.04e-04 |
| *PI16* | 2 | 0.84 | 3.00e-09 | *CAV1* | 2 | 0.63 | 2.74e-09 | *IGFBP5* | 2 | 1.12 | 3.51e-08 | *SOX4* | 3 | 0.54 | 3.23e-04 |
| *ENO1* | 2 | 0.60 | 3.84e-09 | *SERPINE1* | 2 | 0.85 | 5.12e-09 | *CAV1* | 2 | 0.58 | 2.43e-06 | *EHD2* | 4 | 0.51 | 3.76e-04 |
| *TIMP1* | 2 | 0.82 | 4.93e-09 | *TIMP1* | 2 | 0.80 | 4.64e-08 | *CLDN11* | 2 | 0.94 | 4.77e-05 | *TGFBI* | 4 | 1.05 | 8.79e-04 |
| *CAV1* | 2 | 0.58 | 2.96e-07 | *CLDN11* | 2 | 0.96 | 6.37e-07 | *PLA2G2A* | 2 | 1.06 | 2.55e-04 | *S100A10* | 5 | 0.85 | 8.59e-12 |
| *GAPDH* | 2 | 0.58 | 4.41e-07 | *IGFBP5* | 2 | 1.01 | 9.65e-06 | *S100A4* | 2 | 0.70 | 3.62e-04 | *SH3BGRL3* | 5 | 0.84 | 2.30e-09 |
| *SEMA3C* | 2 | 0.83 | 3.13e-05 | *PLA2G2A* | 2 | 1.21 | 1.25e-05 | *TIMP1* | 2 | 0.50 | 6.68e-04 | *S100A4* | 5 | 1.09 | 7.62e-09 |
| *TGFBI* | 2 | 0.91 | 4.35e-05 | *GAPDH* | 2 | 0.56 | 3.94e-05 | *SFRP2* | 3 | 0.56 | 4.74e-05 | *DCN* | 5 | 1.00 | 1.08e-08 |
| *PLA2G2A* | 2 | 1.23 | 6.77e-05 | *S100A4* | 2 | 0.69 | 7.77e-05 | *SOX4* | 3 | 0.54 | 1.32e-04 | *CAV1* | 5 | 0.77 | 4.95e-07 |
| *CLDN11* | 2 | 0.86 | 3.08e-04 | *SEMA3C* | 2 | 0.81 | 4.18e-04 | *MMP16* | 3 | 0.90 | 1.70e-04 | *AXL* | 5 | 0.72 | 7.11e-07 |
|  |  |  |  | *MMP16* | 3 | 0.87 | 1.88e-06 | *TGFBI* | 4 | 0.95 | 9.15e-03 | *CTSK* | 5 | 0.79 | 1.16e-06 |
|  |  |  |  |  |  |  |  |  |  |  |  | *SEMA3C* | 5 | 1.00 | 1.43e-06 |
|  |  |  |  |  |  |  |  |  |  |  |  | *TIMP1* | 5 | 0.70 | 2.43e-05 |
|  |  |  |  |  |  |  |  |  |  |  |  | *CLDN11* | 5 | 1.31 | 3.51e-05 |
|  |  |  |  |  |  |  |  |  |  |  |  | *SULF1* | 5 | 0.83 | 5.92e-05 |
|  |  |  |  |  |  |  |  |  |  |  |  | *TGFBR3* | 5 | 0.70 | 6.30e-05 |
|  |  |  |  |  |  |  |  |  |  |  |  | *ABI3BP* | 5 | 0.77 | 1.79e-04 |
|  |  |  |  |  |  |  |  |  |  |  |  |  |  |  |  |

Table S2: percentage of hASCs (P2) from the three cultures used during the single-cell experiments which adhered to the phenotype: CD44^+^/ CD90^+^/ CD105^+^/CD45^–^/CD34^–^/CD36^+^. All cultures adhered to the phenotypic criteria set out by the International Federation for Adipose Therapeutics and Science (IFATS) and the International Society for Cellular Therapy (ISCT). This table is relevant to the Material and Method section, and Figure S1.

| Culture | Sex | CD44^+^/CD90^+^/CD105^+^/CD45^–^/CD34^–^/CD36^+^ |
| --- | --- | --- |
| A20 | Female | 92.24% |
| A28 | Female | 88.00% |
| A10 | Female | 90.94% |
